# Synthetic Circular gRNA Mediated Biological Function of CRISPR-(d)Cas9 System

**DOI:** 10.3389/fcell.2022.863431

**Published:** 2022-04-04

**Authors:** Mingxia Wang, Jinming Xu, Jialin Meng, Xinbo Huang

**Affiliations:** ^1^ Peking University Shenzhen Hospital, Shenzhen Peking University-The Hong Kong University of Science and Technology Medical Center, Shenzhen, China; ^2^ Department of Dermatology, Institute of Dermatology, Peking University Shenzhen Hospital, Shenzhen Peking University-The Hong Kong University of Science and Technology Medical Center, Shenzhen, China; ^3^ Shantou University Medical College, Shantou, China; ^4^ Department of Urology, The First Affiliated Hospital of Anhui Medical University, Institute of Urology, Anhui Medical University, Hefei, China; ^5^ Anhui Province Key Laboratory of Genitourinary Diseases, Anhui Medical University, Hefei, China

**Keywords:** synthetic biology, CRISPR-(d)Cas9, circular gRNA, gene editing, off-target

## Abstract

Ever since the gene editing function was discovered in the CRISPR-Cas9 system, numerous applications and utilities were investigated in order to apply this technique to medical use. However, the clinical practice was limited by unsatisfactory efficiency and unacceptable off-target editing. Modifications from different aspects of the Cas9 protein and gRNAs were published that aimed to improve its function in one way or another. Under the inspiration of Jacob L. Litke and Samie R. Jaffrey, we propose a novel gRNA design that could achieve rapid circular gRNA assembly inside the cells. This circular design consists of the gRNA of interested flanked by Twister ribozymes. The function of this circular gRNA was proved *in vitro* in both CRISPR-dCas9 and CRISPR-Cas9 systems. It presented a remarkable reduction in the off-target rate in accompany with reduced efficiency. With future improvement in its efficiency, this tool broadens our understanding and possibility of the CRISPR application.

## Introduction

The type II clustered regularly interspaced short palindromic repeats (CRISPR) and its associated protein Cas9 were first discovered in the bacteria and archaea as an immune mechanism against the viral infection (Horvath and Barrangou, 2010; Jinek et al., 2012). It was able to edit the target DNA sequence, and soon became the most popular gene editing tool in the past decade due to its simplicity and high cost-effectiveness (Jinek et al., 2012). CRISPR-Cas9 system is composed of the Cas9 protein and guide RNA (gRNA). Under the guidance of gRNA, the Cas9 protein can bind to and cleave the targeted gene sequence, leaving the DNA double-stranded break (DSB) (Lieber, 2010). This will then stimulate the DNA repair via either homologous recombination or non-homologous end joining (Ran et al., 2013). Except for the editing ability, when mutating RuvC1 and HNH nuclease domains, Cas9 loses its cleavage ability and provides a transcriptional regulatory function which is commonly known as CRISPR-dCas9(Jiang and Doudna, 2017).

Despite it greatly contributing to the progress of gene editing and acts as a potential for future genetic therapy, several characteristics prevent its application in clinical settings. The linear sgRNA once produced intracellularly, cannot last for long. Within 4 h it would be degraded to near-background level, which lowered the CRISPR-Cas9 editing efficiency (Nelson et al., 2021). Cas9 protein could effectively protect the sgRNA in the form of Cas9-sgRNA complex and prolong its half-life. In addition to the short half-life of unbound sgRNA, off-target editing is a more worrying hurdle since this can lead to wrongly edited cells and possibly lead to major side effects (Pawluk et al., 2018).

Modifications aiming to improve the function of CRISPR systems from either the gRNA or Cas9 protein were frequently investigated in the recent decade. In terms of Cas9 modifications, two main improvements were fusing key HDR pathway related modules to Cas9 protein and inducing point mutation to improve the specificity of Cas9 protein (Ma et al., 2020). The first way was good at raising the editing efficiency yet has little contribution to reducing the off-target editing (Shao et al., 2017; Charpentier et al., 2018; Jayavaradhan et al., 2019). The second way was better at specificity yet cannot improve the efficiency (Slaymaker et al., 2016; Chen et al., 2017; Hu et al., 2018). In addition, the fusion protein caused the problem in delivery for future application, since AAV packaging required a strict size limitation (Ma et al., 2020).

Compared to modifying a protein, RNA without a doubt was easier to work with. Though the circular gRNA has never been discussed before, other modifications on RNA monomers such as 2′-O-methyl groups, 2′-F, and phosphorothioate showed increased editing efficiency, improved resistance to nucleases and enhanced stability (Hendel et al., 2015; Rahdar et al., 2015; Yin et al., 2017). Yet these all required solid-phase RNA synthesis. Structural modifications on gRNA have also been proposed, where the 5′ Cap and 3′ PolyA tail was introduced. They could promote editing efficiency and stability (Mu et al., 2019). Hairpin structure on the 3′ end was also included where an increase in the efficiency and stability was observed, accompanied by low toxicity and off-target effect (Nahar et al., 2018).

The promising results gained from previous gRNA modifications inspired us to improve the limitations of the CRISPR system *via* modifying the linear sgRNA into a circular form. Endogenous formation of circular RNAs includes the mRNA ‘back-splicing’ reactions and tRNA splicing (Otsuka et al., 1981; Jeck et al., 2013). Previously, Jacob L. Litke et al. suggested that by expressing the RNAs containing a 5′ hydroxyl and a 2′,3′-cyclic phosphate flanked by Twister ribozymes, they could effectively become substrates for RtcB-mediated ligation after autocatalytic processing (Litke and Jaffrey, 2019). The ligation process resulted in a circular RNA between the ribozymes. This method was utilised in our study to create for the first time the circular sgRNA, and its biological function was examined in both CRISPR-dCas9 and CRISPR-Cas9 systems. In this study, we revealed an effective activation of both CRISPR systems yet at a lower level in the circular gRNA group compared to the linear one. This decrease in efficiency was companied by a substantial decrease in off-target editing.

## Result

### Construction and Verification of Circular gRNA

**FIGURE 1:**
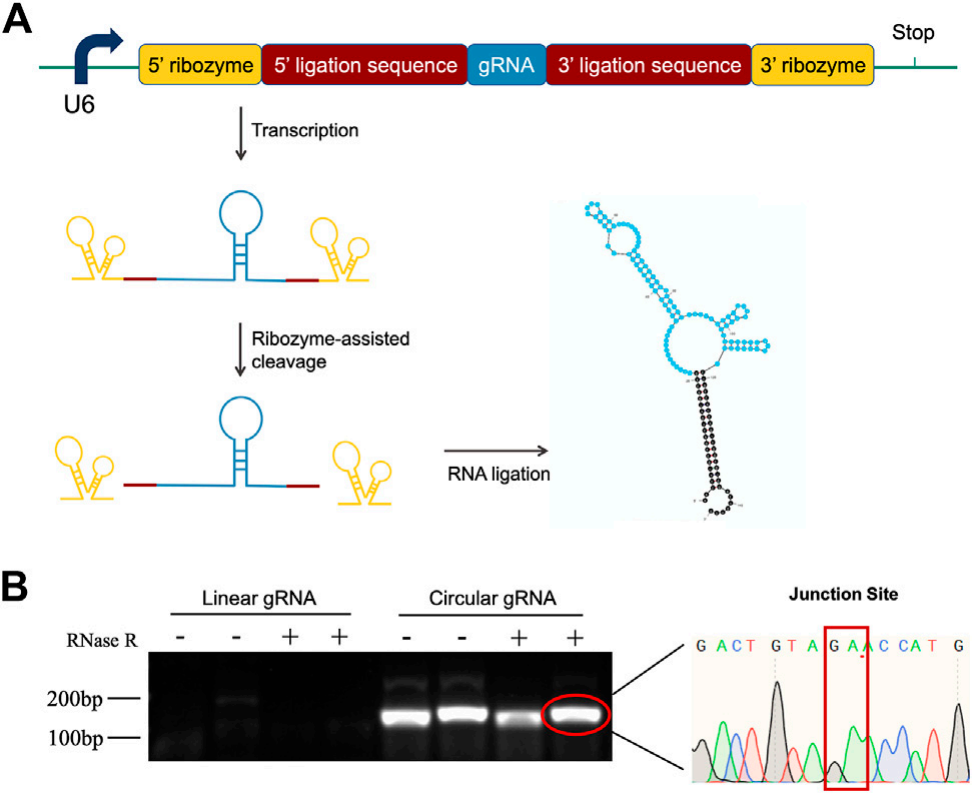
Construction design and validation of circular gRNA. **(A)** The gRNA sequence (blue) is flanked by the 5′- and 3′ligation sequences (brick red). Each of them is linked with 5′- and 3′-self-cleaving ribozymes (yellow). After transcription, the autocleavage ribozymes generate a 2′,3′-cyclic phosphate and 5′-OH on the pre-circular RNA ends, which then were hybridized and formed the circular gRNA by the endogenous RtcB ligase. **(B)** Divergent primers were used to amplify both the circular and linear gRNAs, then the samples were subjected to RNase R digestion. Only the circular gRNA stayed and the product of gel extraction was sent for sequencing. The junction site of the circular hybridization was presented.

To investigate the function of circular gRNA, we constructed a ribozyme-based endogenous-spliced device. The processing of the circular gRNA was demonstrated in Figure 1A, with different colour schemes representing the essential components. We assembled the gRNA according to the design of Jacob L. Litke et al. so that the ribozymes at both ends could cleave the gRNA leaving 3′ and 5′ ligation sequences. The sequences would be hybridized to each other by the endogenous ligase RtcB.

To confirm the transfected plasmid formed circular gRNA, we assessed its sensibility to the RNase R and designed divergent primers for the circular gRNA. The divergent primers could amplify the gRNA sequence and it was not affected by the RNase R degradation. Following the gel electrophoresis, the gel-purified circular gRNA was sent for sequencing. The junction site sequence created by the cleavage of two ribozymes was indicated in Figure 1B.

### Weakened Transcriptional Activating Effect of Circular gRNA on the CRISPR-dCas9

**FIGURE 2:**
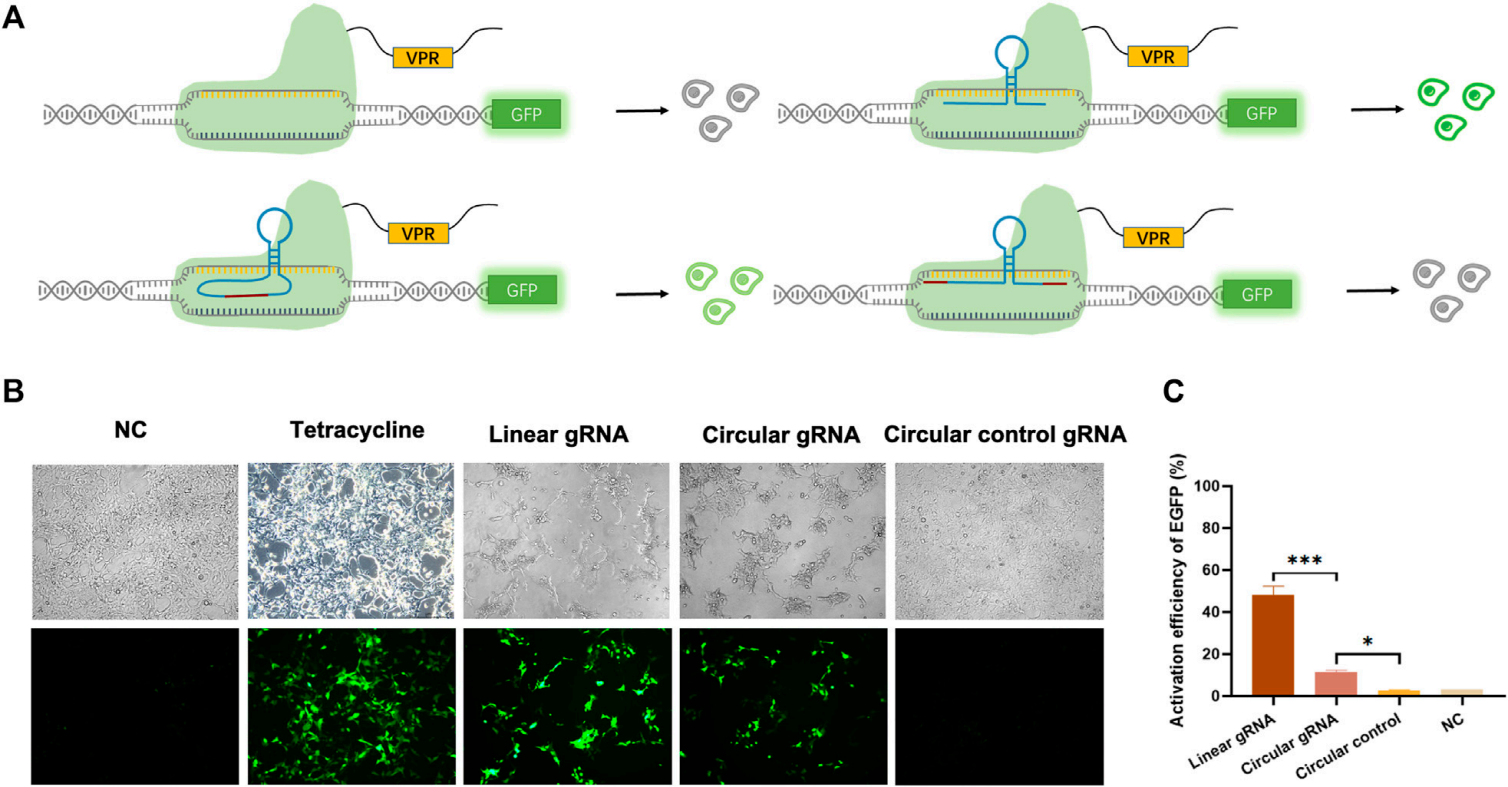
Circular gRNA mediated CRISPR-dCas9 activation. **(A)** A depiction of the CRISPR-dCas9 system consisted of TRE targeted gRNAs, including a linear gRNA, circular gRNA or circular control gRNA, and a dCas9-VPR protein. The stably transfected 293FT-411 cell line contained an EGFP protein driven by the TRE promoter. Green fluorescent was recorded as a reference of the efficiency of transcriptional activation by different gRNAs. **(B)** Representative microscopy images displaying the fluorescence intensity and frequency of EGFP 48 h after transfection. **(C)** Quantitative measurement of EGFP expression via qPCR 48 h after transfection. Data were normalized to tetracycline activated EGFP expression, which is regarded as 100%. Data are means ± SD from three independent experiments (*p < 0.05, ***p < 0.001).

To validate whether the circular gRNA performs its guiding function and forms complex with dCas9 protein, we transfected the HEK293T-411 cells with the CRISPR-dCas9 system. A linear gRNA group was included as a positive control. The CRISPR-dCas9 system is composed of the TRE promoter targeting gRNA and a dCas9-VP64. The HEK293T-411 cell line was stably transfected with EGFP genes with a TRE promoter. Without activating the promoter, the cells remained dim (Figure 2A). To eliminate the effect of possible unsuccessful circular gRNAs formed during the ligation process with a linear gRNA flanked by the 3′ and 5′ ligation sequence, negative control of circular gRNA was added. It had the same 3′ and 5′ ligation sequence but no ribozyme was attached beside them, thus, it couldn’t form a circular structure inside the cells.

The linear gRNA could guide the dCas9-VP64 to the TRE promoter region and activate the downstream EGFP expression, resulting in bright green light (Figure 2B). The circular gRNA could also perform the guiding function, but at a much lower efficiency compared to the linear gRNA. Yet, a substantial amount of EGFP was expressed to reveal a detectable green light (Figure 2B). There was no difference observed in the green fluorescence between the negative circular gRNA control group and the control with no gRNA introduced (Figure 2B). This trend in EGFP fluorescence activation is further supported by our flow cytometry results (Supplementary Figure S1). Other than measuring fluorescence, the quantitative qPCR was utilised to reveal the relative mRNA expression level of EGFP. Results suggested that the EGFP expression was 48.1% under the guidance of linear gRNA when standardised to the level of tetracycline activation. This was significantly outperformed the circular gRNA, which activated the expression of about 11.4% EGFP genes (Figure 2C). However, the activation level of circular gRNA was statistically higher than the circular gRNA control or the negative control. A weakened yet certain transcriptional activating effect was observed in the circular gRNA when compared to the linear one.

### Reduced CRISPR-Cas9 On- and Off-Target Editing of Circular gRNA

**FIGURE 3:**
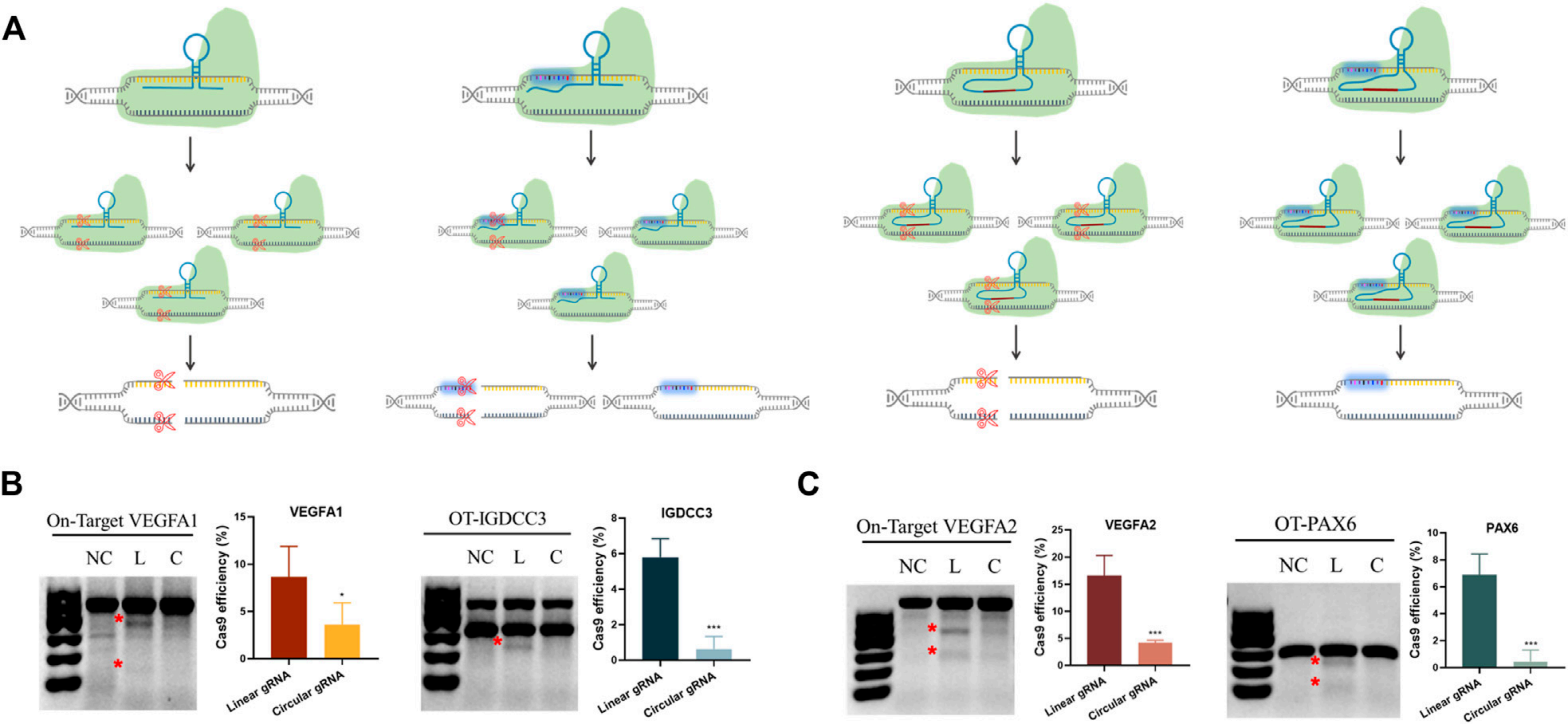
Reduced CRISPR-Cas9 efficiency and off-target rates in the circular gRNA compared to the linear gRNA. **(A)** A depiction of CRISPR-Cas9 targeted DNA cleavage under the guidance of linear and circular gRNA. The linear gRNA allowed efficient on-target editing accompanied by some off-target events. The circular gRNA showed a lower rate of on-target editing and the off-target events were largely abolished. **(B,C)** The representative results of the T7E1 assay on the on- and off-target editing rates in linear and circular gRNA. Data were standardised to the no gRNA negative control group, and were presented as means ± SD from at least three independent experiments (*p < 0.05, ***p < 0.001).

Based on the transcriptional regulatory results, we knew the circular gRNA could form a complex with Cas9 protein. Yet, whether this complex was stable enough for Cas9 protein to perform its gene editing role required further validation. The transfection of either linear or circular gRNA with Cas9 proteins to the HEK293T cells was carried out, so that a direct comparison about editing efficiency and off-target rate between the two gRNAs could be made (Figure 3A).

Two different sites of the VEGFA gene were selected as the targets. The off-target sites of VEGFA site1 were the PAX6 gene, and the off-target site of VEGFA site2 was IGDCC3. T7E1 analysis showed that both gRNAs could lead the Cas9 protein to target DNA and perform the gene editing role (Figure 3B). From the semi-quantitative analysis, linear gRNA had significantly higher efficiency compared to the circular gRNA, and this applied to both the on-target and off-target editing (Figures 3B,C). The editing efficiency of VEGFA site one and site two was 8.6% and 16.6% respectively under the guidance of linear gRNA, while it was 3.6% and 4.2% in the group of circular gRNAs (Figure 3B). Worth mentioning, the cleavage of both off-target genes was almost abolished in the circular gRNA groups, yet the linear gRNA groups showed off-target rates higher than 5% (Figure 3C). This implied a more specific inhibition of circular gRNA on the off-target editing.

### Enhanced Stability of Circular gRNA Guided Cas9 Cleavage

**FIGURE 4:**
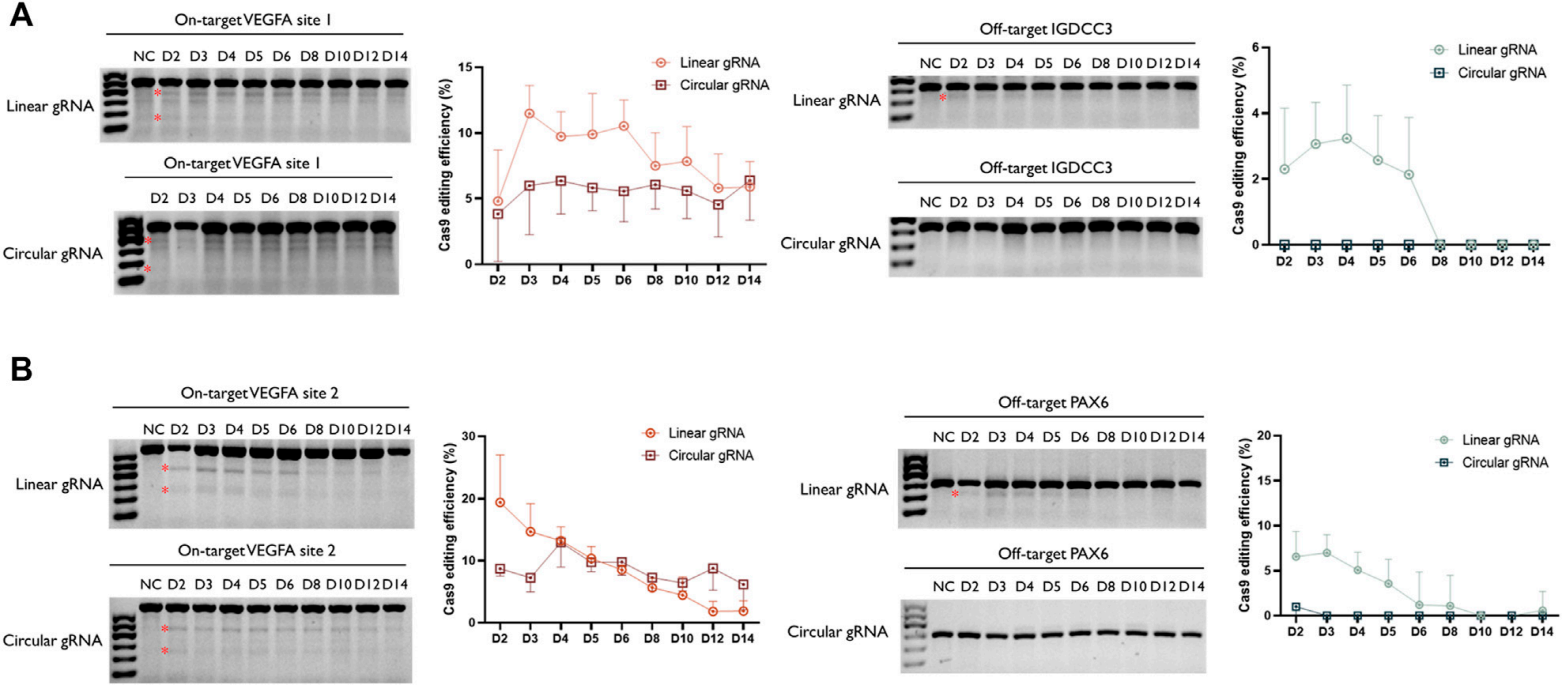
Enhanced stability of circular gRNA guided Cas9 cleavage. Representative T7EI assay for visualization of VEGFA1 **(A)** and VEGFA2 **(B)** on- and off-target editing. The genomic DNA was collected from the transiently transfected samples from day 2 to day 14. The semi-quantitative analysis of the T7E1 assay further confirmed the ability of both gRNAs to facilitate the Cas9 cleavage. In addition, through the time-course assessment, a more stable performance was observed in the group of circular gRNA. The relative Cas9 editing efficiencies were standardised to the no gRNA negative control group. Data were means ± SD from three independent experiments.

Following the validation of its CRISPR-Cas9 editing function, we wished to examine whether the stability of circular gRNA could exceed the linear one. Through a two-week-time transfection, the circular gRNA showed a lower rate of editing efficiency in the VEGFA1 site, and the editing level was maintained more stable than the linear one. Throughout the period, the editing efficiency of the circular gRNA group was approximately 5.7% whereas, in the linear gRNA group, it dropped roughly from 11.5% to 5.9% (Figure 4A). The comparison between linear and circular gRNA at the VEGFA2 site was more apparent. The efficiency of editing of linear gRNA was higher during the first 5 days of transfection, yet from day 6 the declining trend of editing efficiency in the linear gRNA group caused an overall lower efficiency compared to the circular gRNA group. Throughout the period, the editing efficiency of the circular gRNA group was approximately 8.6% whereas, in the linear gRNA group, it dropped roughly from 19.4% to 1.8% (Figure 4B).

Besides the editing efficiency, the off-target rate was also examined. Surprisingly, the circular gRNA group almost wiped off the off-target editing of both targets. For VEGFA1, the linear gRNA group had approximately 2.7% editing efficiency on its off-target IGDCC3 gene in the first 4 days, and soon the editing declined to 0% at day 8 (Figure 4A). This editing was undetectable in the circular gRNA group. For VEGFA2, the linear gRNA group had a steady editing efficiency of 6.2% on its off-target PAX6 in the first 3 days, and soon the editing declined to 0% on day 10. The circular gRNA group only showed around 1.0% editing on the off-target at day 2 and this off-target editing became undetectable afterward (Figure 4B).

## Discussion

In this study, we for the first time designed the circular gRNA and confirmed its biological function in the CRISPR system *in vitro*. By adding ribozymes at both 3′ and 5′ ends of the gRNA, the auto-cleavage would happen after the plasmid transcription and endogenous ligase RtcB21 could hybrid both ends. The circular gRNA could also perform the guiding function in the dCas9 system, but at a lower efficiency compared to the linear gRNA. The editing efficiency of circular gRNA on target genes was not as efficient, but at greater stability. In our study, the circular gRNA group almost wiped off the off-target editing of both targets.

The compromised editing efficiency and the off-target effect of circular gRNA may be due to the structural change between the linear and circular gRNA altered its capacity to be complimentary to Cas9 protein dropped. Except for these two aspects, the sustainability of circular gRNA was also analysed. Numerous studies have proposed a fold increase in stability of *in vitro* RNAs in circular rather than linear form (Hsiao et al., 2017). Linear RNAs were prone to degradation, and usually only last for a few hours (Filonov et al., 2015). Circular RNAs on the other hand could last for days intracellularly (Han et al., 2018).

Initially, we anticipated the prolonged half-life of circular gRNA would allow a prolonged effect in both the CRISPR-dCas9 system and CRISPR-Cas9 system, which could be beneficial for future applications. The prolonged effect of circular gRNA was supported with our Cas9 T7E1 endonuclease assay, where a more rapid decrease in the editing efficiency was observed in the linear group compared to the circular one. Yet, our results indicated the duration of dCas9 regulated EGFP expression did not sustain longer in the circular group (not shown in the figure). This may be due to the good stability of our reporter fluorescence. In the past, the degeneration period of EGFP as a reporter was about 5–10 days (Yuen et al., 2017). With the initial larger amount of EGFP protein being produced in the linear group, the stability of EGFP failed to act as a prompt reporter for the status of gRNAs. In addition, linear gRNA has a stable binding to (d)Cas9 protein. The circular gRNA may weaken the natural chimeric ability with Cas9 protein to some extent due to its cyclization structure. Also, the transformation from a linear process to a cyclized structure requires an intermediate processing process, which may weaken the efficiency of the system to some extent.

In summary, our study designed a circular gRNA and confirmed its biological function in the CRISPR system *in vitro*. Currently, its efficiency remained a drawback compared to the traditional linear gRNA, which could be further investigated and improved in future studies. Yet this adventurous design of gRNA offers the novel possibility of CRISPR applications and widen our toolset of CRISPR.

## Materials and Methods

### Design of Circular gRNA

In this study, we designed a cyclized gRNA in the form of “Tornado” system inspired by Jacob L. Litke et al. We use the connection sequences to connect Twister P1 and Twister P3 U2A to both ends of the gRNA.

3’ stem-forming sequence： aaccatgccgactgatggcag.

5’ stem-forming sequence： ctgccatcagtcggcgtggactgtag.

Twister U2A ribozyme cDNA sequence:

gcc​atc​agt​cgc​cgg​tcc​caa​gcc​cgg​ata​aaa​tgg​gag​ggg​gcg​gga​aac​cgc​ct.

P1 Twister ribozyme cDNA sequence:

aac​act​gcc​aat​gcc​ggt​ccc​aag​ccc​gga​taa​aag​tgg​agg​gta​cag​tcc​acg​c.

### Plasmid Construction

Each plasmid used for CRISPR-dCas9 assessment contained both the target-corresponding gRNA sequence and the dCas9-VPR sequence. The linear gRNA vector, circular control gRNA vector and circular gRNA vector in this study were constructed by Syngentech Co., Ltd. (Beijing, China). The modified gRNAs were driven by the U6 promoter and the dCas9 protein was driven by the pHEf1A promoter. The plasmids involved for CRISPR-Cas9 assessment were co-transfected as separate plasmids for gRNA and Cas9 protein. The linear and circular gRNAs were driven by the U6 promoter and the Cas9 protein was driven by the pHEf1A promoter. The relative sequences were listed in Supplementary Table S1. The above plasmid abbreviations were listed in Supplementary Table S2.

### Cell Lines and Cell Culture

HEK293T and HEK293FT-411 cells were included in this study, and the HEK293T-411 cells were HEK293T cells stably transfected with TRE-promoted EGFP gene. Both cell lines were cultured in the DMEM medium (Invitrogen, United States) that was supplemented by 10% fetal bovine serum (Gibco, United States), 100 μg/ml streptomycin and 100 U/mL penicillin. The cells were placed in the 37°C, 5% CO2 incubator.

### Cell Transfection

When the plated cells reached 70–80% confluency, Lipofectamine 3,000 (Invitrogen, United States) would be used to transiently transfected the plasmid according to the manufacturer’s protocol. The transfected cells were harvested after 48 h using trypsin. With HEK293FT-411cells, the fluorescence of EGFP was measured by fluorescent microscope (Leica DMi8 C, Germany) and RT-qPCR (LightCycler^®^ 480 II, Roche, Switzerland). With HEK293T cells, their genomic DNA was extracted and the editing efficiency of related genes was measured by PCR and T7 endonuclease I assay.

### RNA Extraction and RNase Assay

After 48 h of transfection, total RNA was extracted following the manufacturing protocol using Total RNA Isolation Kit (Beibei Biotech, China) and collected in RNase-free Eppendorf. The RNA concentration was determined by Nanodrop (Thermo Scientific, United States) and then stored in −80°C.

Rnase assay was applied to the total RNA collected. In 1 µg of total RNA, 1 µl of 10x reaction buffer and 0.15 µl RNase R (Geneseed Biotech, China) were added. RNase-Free water would supplement the system to 10 µl. The digestive activity of the enzyme would be maintained at 37°C for 15 min, followed by 10 min denaturation at 70°C.

The product of RNase assay would be subjected to reverse transcription using All-in-One First-Strand cDNA Synthesis SuperMix for qPCR (One-step gDNA Removal) (TransScript, China) according to the manufacturing protocol. Then the cDNA would be ready for PCR under the protocol (95°C for 3 min, 30 cycles at 98°C for 10 s, 53°C for 30 s, 72°C for 18 s, then 72°C for 5 min and cooled down to 4°C) by EmeraldAmp PCR Master Mix (2x Premix) (TaKaRa, Japan). The PCR products were sent to Sangon Biotech for Sanger sequencing afterward. The primers used to check the circular gRNA were:

Forward primer: 5 ′-GGC​TAG​TCC​GTT​ATC​AAC​TTG-3′.

Reverse primer: 5′-CTA​TTT​CTA​GCT​CTA​AAA​CTA​TCA​GTG-3′

### qPCR

In this experiment, HEK293FT-411 was transfected with the linear gRNA vector, circular control gRNA vector or circular gRNA vector targeting the promoter region of EGFP gene. Then, the total RNA was harvested from the cells and cDNA was synthesized according to the previous protocols. qPCR was used for quantitative analysis of the target gene expression and the relative EGFP mRNA expression was assessed by SYBR Green qPCR MasterMix (Takara, Dalian, China). GAPDH acted as the control and the relative quantity was calculated by △△Ct method. The primers for GAPDH and EGFP were listed below in 5′ to 3′ direction:

GAPDH (F): TCC​CAT​CAC​CAT​CTT​CCA.

GAPDH (R): CAT​CAC​GCC​ACA​GTT​TCC.

EGFP (F): ACG​ACG​GCA​ACT​ACA​AGA​CC.

EGFP (R): TTG​TAC​TCC​AGC​TTG​TGC​CC.

### Flow Cytometry Assay

In this experiment, we used the FITC channel to detect cell fluorescence intensity. HEK293FT-411 cells were digested with trypsin without EDTA and washed twice with PBS before resuspended for the flow cytometry. The instrument used in this experiment is the Flow Cytometer (Epics, XL-4, Beckman, CA, United States). HEK293FT-411 cells without transfection were included to identify the background EGFP expression level. Then the collected samples were divided into EGFP positive and negative groups.

### PCR and T7 Endonuclease I Analysis

After 48 h of transfection, the genomic DNA of HEK293T cells was extracted by the Ultra DNA Isolation Kit (Bei-Bei Biotech). In each 20 µl PCR system, 100 ng genomic DNA was added as a template. EmeraldAmp PCR Master Mix (2x Premix) (TaKaRa, Japan) was used to amplify the DNA under the standard protocol (95°C for 3 min, 30 cycles at 98°C for 10 s, 54°C for 30 s, 72°C for 35 s, 72°C for 5 min and cooled down to 4°C).

For the T7E1 assay, each 10 µl reaction system included 3 µl PCR products, 6 µl distill water and 1 µl 10x T7E1 Reaction Buffer. The standard denaturing/annealing process was illustrated as followed: 95°C for 5 min, 95°C–85°C for −2°C/s, 85–25°C for −0.1°C/s, 25°C for 5 min and cooled down to 4°C. Following this, 0.25 µl T7E1 enzyme (Betotime) was applied to the mix to cleave the mismatched DNA sequences. The incubation lasted 25 min at 37°C. Then, the cleavage was halted by heating to 85°C for 10 min. Final products underwent gel electrophoresis on 1% agarose gel, the target and cleaved bands were quantitated using ImageJ. The indel rate was calculated using the following formula: % gene modification = 100 x (1—(1- fraction cleaved)^1/2^) (Guschin et al., 2010). The primers used for VEGFA target site 1 and 2, and their respective off-target sites IGDCC3 and PAX6 were validated in previous studies (Fu et al., 2013) and listed below, with directions ranging from 5′to 3’:

VEGFA1 (F): AGA​GAA​GTC​GAG​GAA​GAG​AGA​G.

VEGFA1 (R): CAG​CAG​AAA​GTT​CAT​GGT​TTC​G.

VEGFA2 (F): TCC​AGA​TGG​CAC​ATT​GTC​AG.

VEGFA2 (R): AGG​GAG​CAG​GAA​AGT​GAG​GT.

IGDCC3 (F): ACC​CCA​CAG​CCA​GGT​TTT​CA.

IGDCC3 (R): GAA​TCA​CTG​CAC​CTG​GCC​ATC.

PAX6 (F): CAA​GAT​GTG​CAC​TTG​GGC​TA.

PAX6 (R): GCA​GCC​TAT​TGT​CTC​CTG​GT.

### Statistical Analysis

All experimental data were calculated as the means ± standard deviation (SD). Measurements were collected from at least three independent experiments. One-way ANOVA analysis was applied and *p* < 0.05 was considered as statistically significant.

## Data Availability

The original contributions presented in the study are included in the article/Supplementary Material, further inquiries can be directed to the corresponding author.
