# Supplementary material for: Synthetic Circular gRNA Mediated Biological Function of CRISPR-(d)Cas9 System
Source: Front Cell Dev Biol. 2022 Apr 4;10:863431. doi: 10.3389/fcell.2022.863431 (PMC9013764; doi:10.3389/fcell.2022.863431)
Supplement: Supplementary file 1 [file DataSheet1.docx]

### Synthetic circular gRNA mediated biological function of CRISPR-(d)Cas9 system

Mingxia Wang et al.

**Supplementary Information**

**Supplementary Table.1 Relative sequences used in this study.**

| Name | cDNA sequence |
| --- | --- |
| Circular gRNA Forward primer | 5 ′-GGCTAGTCCGTTATCAACTTG-3′ |
| Circular gRNA Reverse primer | 5′-CTATTTCTAGCTCTAAAACTATCAGTG-3′ |
| GAPDH (F) | TCCCATCACCATCTTCCA |
| GAPDH (R) | CATCACGCCACAGTTTCC |
| EGFP (F) | ACGACGGCAACTACAAGACC |
| EGFP (R) | TTGTACTCCAGCTTGTGCCC |
| VEGFA1 (F) | AGAGAAGTCGAGGAAGAGAGAG |
| VEGFA1 (R) | CAGCAGAAAGTTCATGGTTTCG |
| VEGFA2 (F) | TCCAGATGGCACATTGTCAG |
| VEGFA2 (R) | AGGGAGCAGGAAAGTGAGGT |
| IGDCC3 (F) | ACCCCACAGCCAGGTTTTCA |
| IGDCC3 (R) | GAATCACTGCACCTGGCCATC |
| PAX6 (F) | CAAGATGTGCACTTGGGCTA |
| PAX6 (R) | GCAGCCTATTGTCTCCTGGT |
| Twister U2A ribozyme | gccatcagtcgccggtcccaagcccggataaaatgggagggggcgggaaaccgcct |
| P1 Twister ribozyme | aacactgccaatgccggtcccaagcccggataaaagtggagggtacagtccacgc |
| 3’ stem-forming sequence | aaccatgccgactgatggcag |
| 5’ stem-forming sequence | ctgccatcagtcggcgtggactgtag |

**Supplementary Table.2 Maps of plasmids used in this study.**

| pHS-ACR-LW998 | pZDonor_U6-gRNA(KG)-hEF1a-NLS-dCas9-NLS-VPR |
| --- | --- |
| pHS-ACR-LY070 | pZDonor_U6- RNA-VEGFA gRNA-1-gRNA backbone-RNA-hEF1a-Puro |
| pHS-BVC-LJ007 | pZDonor_U6-gRNA(TRE)-hEF1a-NLS-dCas9-NLS-VPR |
| pHS-ACR-LY071 | pZDonor_U6- RNA-VEGFA gRNA-2-gRNA backbone-RNA-hEF1a-Puro |
| pHS-ACR-LW042 | pZDonor_hU6-Neg sgRNA-backbone-hEF1a-NLS-dCas9-NLS-VPR |
| pHS-ACR-0200  (cirlular control gRNA) | pZDonor_U6-tRNA-TRE gRNA-gRNA backbone-tRNA-hEF1a-NLS-dCas9-NLS-VPR |

**Supplementary Figure.1**

**
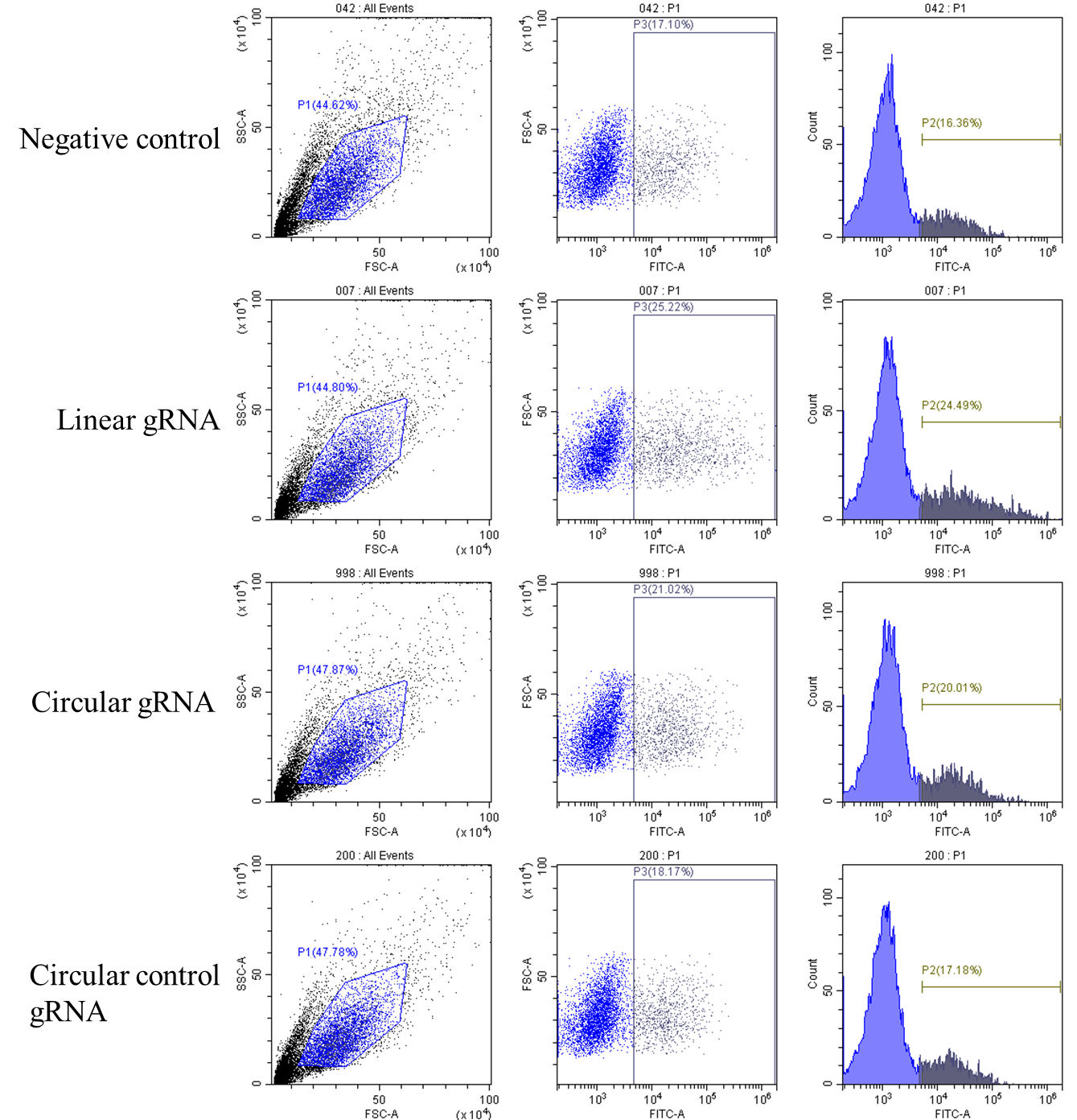
**

**Supplementary Figure 1. Percentage of EGFP activation mediated by CRISPR-dCas9 system in linear and circular gRNA groups.** Representative flow cytometry images displaying the fluorescence intensity of EGFP in different groups, including negative control, linear gRNA, circular gRNA and circular control gRNA, 48 hours after transfection.
